# Single-Stage Resection and Reconstruction of Intraosseous Meningioma: The First Reported Case at Our Hospital

**DOI:** 10.7759/cureus.102468

**Published:** 2026-01-28

**Authors:** Martha E Abarca, Lagree G Reynoso, Mauricio A Matus

**Affiliations:** 1 Neurosurgery, Hospital Militar Escuela Dr. Alejandro Dávila Bolaños, Managua, NIC; 2 Surgical Anatomy, Hospital Militar Escuela Dr. Alejandro Dávila Bolaños, Managua, NIC; 3 Neurosurgery, Hospital Militar Escuela Dr. Alejandro Davila Bolaños, Managua, NIC

**Keywords:** case report, cranoplasty, initial treatment, intraosseous meningioma, management, one-stage surgery, titanium mesh cranioplasty, tumor resection

## Abstract

In neurosurgery services worldwide, the resection of benign and malignant tumors continues to pose a challenge due to the need to preserve neurological function, especially when these lesions are located in eloquent areas. Among central nervous system oncologic lesions, meningiomas are the most common. Based on the criteria of the World Health Organization, they are classified into three grades according to their malignancy. In this report, we present the case of a 46-year-old female patient with no relevant medical history, who had experienced headaches for a year, which intensified 24 hours prior to her visit to the emergency department, without aggravating or relieving factors. Physical examination revealed no neurological deficits. A contrast-enhanced computed tomography (CT) scan showed a frontoparietal bony prominence measuring 4.41 x 6.2 cm. Due to its size, a wide surgical resection was performed, followed by histopathological analysis, which confirmed the diagnosis of a primary intraosseous meningioma, WHO grade 1. To repair the bone defect, a cranioplasty with a low-profile titanium mesh was performed.

At the 12-month follow-up, the patient presented with full recovery and resolution of symptoms, with no evidence of recurrence. This case represents a rare entity with no previous reports in Nicaragua, which may be due to its low incidence and limited awareness among the medical community, often leading to underdiagnosis. Since this is the first case report in this institution, we consider it important to document and share it, aiming to contribute to the development of a diagnostic and therapeutic guideline for low-resource settings and developing countries.

## Introduction

The most common primary tumors of the central nervous system are meningiomas, accounting for more than one-third of all oncological lesions in the specialty [1]. According to histological types, they are divided into three grades and are primarily considered benign lesions. Meningiomas predominantly grow from subarachnoid meningothelial cells, occasionally migrating to bone tissue, where they give rise to intraosseous meningiomas. The extradural growth of these tumors is rare. In bone tissue, they have an incidence of less than 2%, with the most common locations being the orbital cavity, frontal bone, and parietal bone [2]. The most common differential diagnoses are primary bone tumors, metastatic lesions, and fibro/osseous lesions. Epidemiologically, intraosseous meningiomas most commonly affect adults between 40 and 80 years of age, with a female predominance. Clinically, patients may present with nonspecific symptoms, most commonly pain that is localized, persistent, and of insidious onset, and progressively worsens as the lesion enlarges. Depending on the location, other manifestations such as swelling or visual disturbances may also occur.

Imaging plays a critical role in the diagnosis and surgical planning of intraosseous meningiomas. Computed tomography (CT) typically demonstrates hyperostosis or lytic bone lesions, while magnetic resonance imaging (MRI) can help delineate the extent of the lesion, its relationship with adjacent dura or soft tissue, and any intracranial involvement. These imaging features are essential for differentiating intraosseous meningiomas from other primary or metastatic bone lesions.

The treatment of choice in these cases is total surgical resection of the lesion, including 1 to 5 mm beyond the periphery, with a recurrence risk of up to 0%, and no need for adjuvant therapy (radiation/chemotherapy) [3]. In low-resource settings, single-stage surgical repair is often preferred over staged approaches, as it reduces overall costs, shortens hospital stay, minimizes the risk of defects from reinterventions, and is more convenient and comfortable for the patient. Surgical planning must consider reconstruction options, particularly in centers that perform alloplastic grafting, to restore structural and cosmetic integrity.

For researchers, the case of intraosseous meningioma is of particular relevance, as no literature has been found to date describing this pathology in the country. Furthermore, it is worth noting that this hospital center is the only one in the country that performs reconstructions using alloplastic grafts. Therefore, its documentation is of great importance in the national context, serving as a valuable reference for the clinical approach and management of this rare condition. The objective of this report is to describe the clinical presentation, radiological features, and surgical management of an intraosseous meningioma in our setting, highlighting considerations relevant to both diagnosis and reconstruction in resource-limited environments.

## Case presentation

A 46-year-old female patient with no personal pathological history presented to the emergency department with a holocranial headache of more than one year’s duration. The pain had recently intensified over the last 24 hours prior to admission, reaching a severity of 10/10. The headache was non-radiating, with no identified alleviating or aggravating factors and no associated symptoms. Upon further questioning, the patient reported a history of minor head trauma one year prior, after which she noticed a bony prominence in the right frontoparietal region.

Neurological examination revealed no deficits. However, a firm, painful lesion was palpable in the right frontoparietal area, measuring approximately 4 x 5 cm in its anteroposterior and mediolateral axes. The lesion was non-pulsatile and immobile, without warmth, erythema, or discharge.

**Figure 1:**
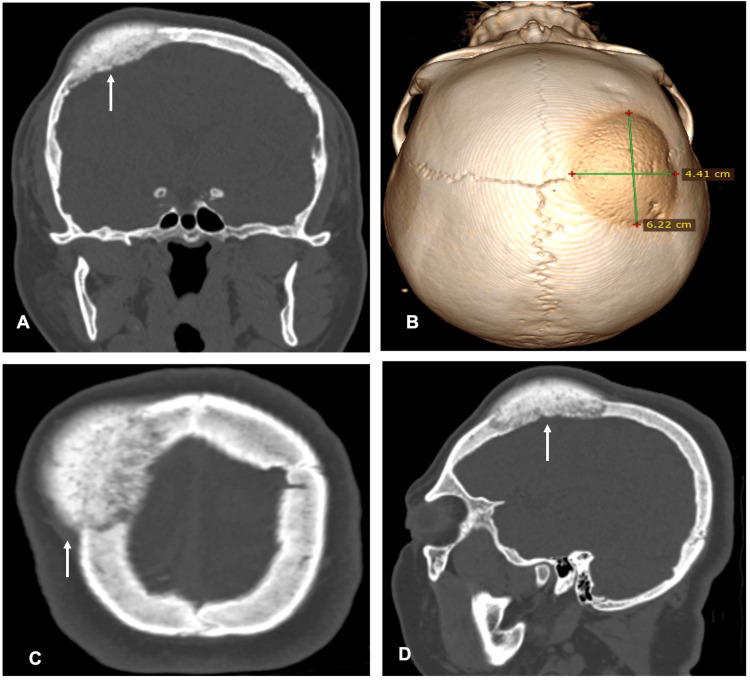
Cerebral computed tomography showing an osteoblastic lesion in the left frontoparietal region, with cortical thickening protruding beyond the margins of the adjacent normal bone. A. Coronal view. B. A 3D reconstruction from cerebral tomography, with measurements performed. C. Axial view. D. Left sagittal view.

As part of the initial approach for headache with red flag features, laboratory tests were ordered to rule out electrolyte or metabolic disturbances, which were subsequently excluded as all results were normal. A brain CT scan was also performed with the aim of ruling out lesions that would require emergency neurosurgical management. The scan revealed an osteoblastic lesion in the right frontoparietal region, measuring 6.22 x 4.41 cm in its anteroposterior and laterolateral diameters, respectively, confined to the bone surface (Figure 1).

**Figure 2:**
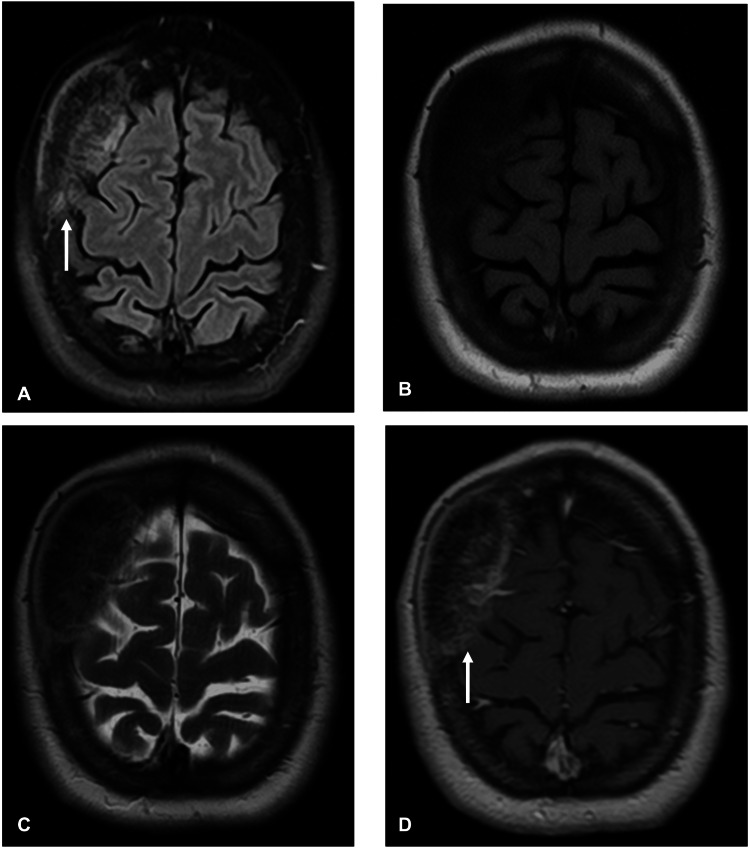
Contrast brain MRI. A. FLAIR image representing hyperintensity and thickened part of the affected skull in comparison to adjacent bone tissue. B. T1-weighted image showing no hyperintensity without contrast administration C. T2-weighted image showing isointensity D. T1-weighted contrast image showing contrast enhancement in a well-defined lesion, with well-delineated margins.

The patient was admitted for further diagnostic evaluation and pain management, as the headache was of sudden onset, and the observed lesion was not deemed sufficient to explain it. She was evaluated by neurology, which suggested that the patient had a coexisting chronic daily headache, which was considered the likely cause of her pain. Both contrast-enhanced and non-contrast brain MRI studies were obtained, showing no evidence of dural, vascular, or cerebral parenchymal infiltration (Figure 2).

Based on the imaging findings, the main differential diagnoses were intraosseous meningioma and fibrous dysplasia. This was due to the osteoblastic nature of the lesion, meaning that the bone was abnormally thickened or overgrown, as opposed to a lytic lesion, where the bone is being destroyed. On MRI, the lesion showed contrast enhancement, which raised suspicion for an intraosseous meningioma. However, no “dural tail” was observed - a feature often seen in typical meningiomas where the covering membrane of the brain (the dura) appears thickened and enhances on imaging; its absence does not rule out the diagnosis but is noteworthy. Fibrous dysplasia was considered because it can also cause bone thickening, but it typically has a “ground-glass” appearance on CT and usually does not enhance with contrast on MRI. Osteoma, osteoblastic metastasis, and Paget’s disease were deemed less likely: osteomas are usually small, well-circumscribed, and asymptomatic; osteoblastic metastases often present with a known primary tumor and may be multiple; and Paget’s disease usually affects multiple bones and occurs in older patients. The brain parenchyma itself was normal.

Surgical resection was indicated as the first-line approach for both definitive histopathological diagnosis and therapeutic purposes. After completing preoperative evaluations and being deemed fit for surgery, the patient underwent a planned procedure for safe maximal resection of the lesion, followed by immediate reconstruction of the resulting cranial defect using a low-profile titanium mesh.

**Figure 3:**
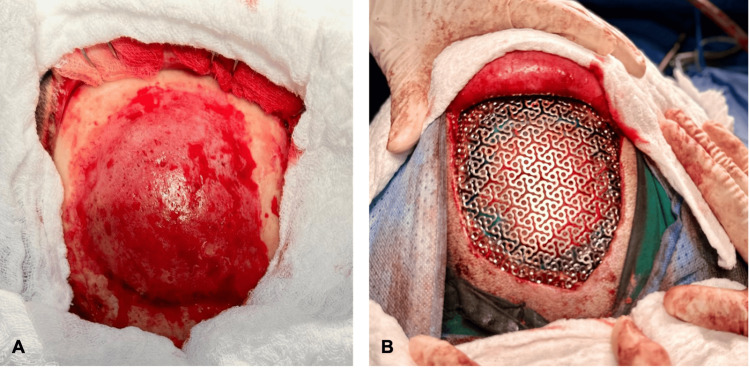
Intraoperative images. A. Intraosseous meningioma in the right frontoparietal region, showing high vascularity, elevated borders, and well-defined margins compared to healthy white colored tissue. B. Cranial defect reconstruction using a low-profile titanium mesh after having removed the affected part of the skull.

Under neuroanesthesia, with the patient in a supine position and the head rotated 45 degrees to the left, surgical planning was performed using craniometry and neuronavigation. A maximal resection was completed, including a 1-cm margin of healthy tissue around the lesion. The surgery proceeded as expected, with an estimated blood loss of 400 mL, no transfusion required, and no intraoperative complications (Figure 3). Subsequently, a 20 x 20 cm titanium mesh was shaped and adapted to the cranial defect, preserving the patient’s cranial anatomy. The mesh was fixed using titanium microplates and microscrews (Figure 3).

The procedure concluded uneventfully. The patient remained hospitalized for 48 hours for postoperative monitoring and care, after which she was discharged with complete resolution of symptoms, no neurological deficits, and a Karnofsky Performance Status score of 100.

At the two-week follow-up, histopathological analysis of the surgical biopsy confirmed a WHO grade I intraosseous meningioma. Complete resection was achieved, with no residual lesion; therefore, adjuvant therapy (radiotherapy or chemotherapy) was not considered necessary. Conservative follow-up with periodic imaging studies was recommended, CT scan imaging was performed at six months after surgery, and no changes were observed.

**Figure 4:**
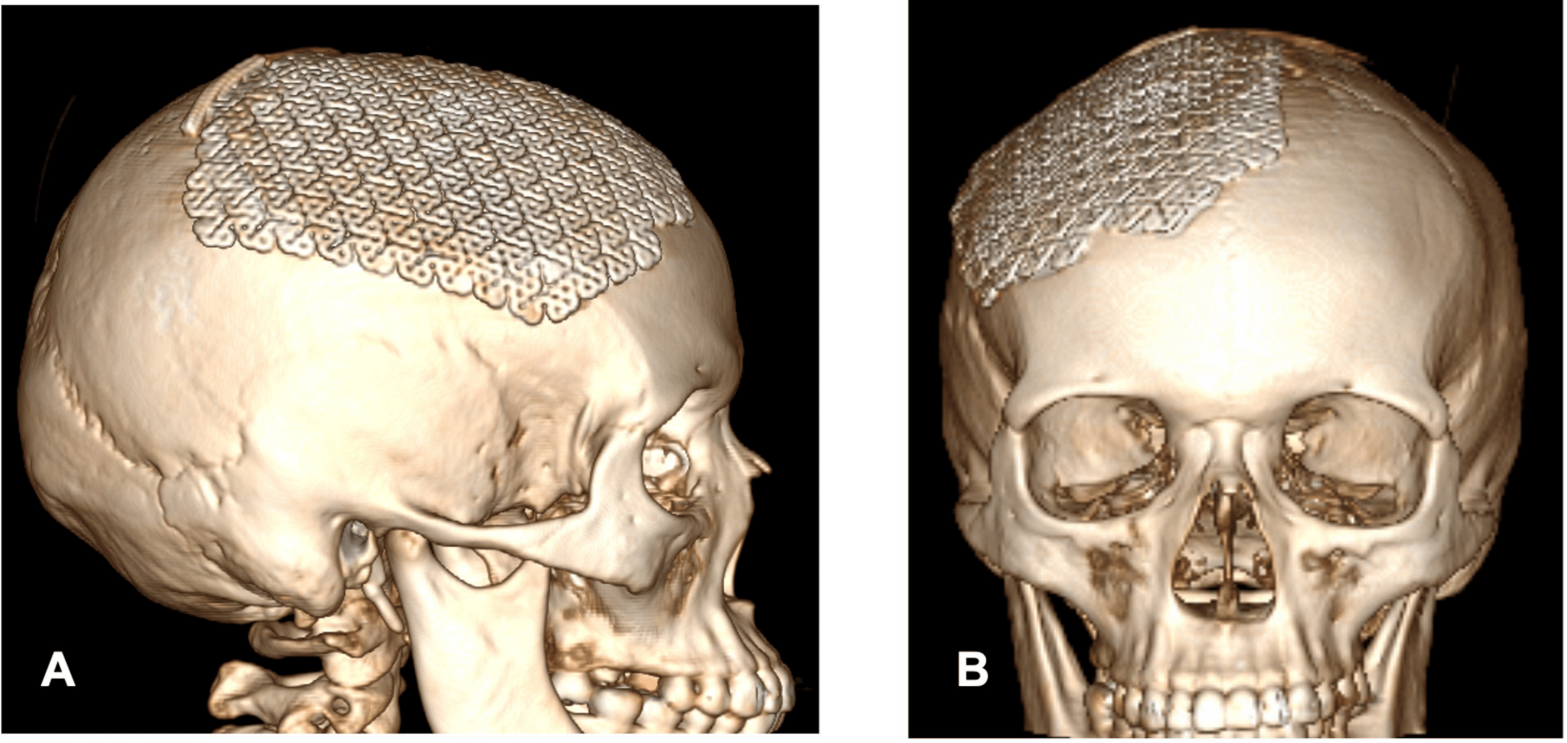
A 3D reconstruction of postoperative CT scan following cranioplasty with low-profile titanium mesh for the correction of cranial defect while preserving the patient's anatomy as observed above. Symmetry was achieved to look as the contralateral side. A. Sagittal view. B. Coronal view.

At the 12-month postoperative follow-up, imaging showed no evidence of tumor recurrence (Figure 4).

## Discussion

Meningioma, also referred to as a tumor of the meninges, is a benign neoplasm that typically exhibits slow growth. It originates from arachnoid cells, a subtype with high metabolic activity involved in the reabsorption of cerebrospinal fluid (CSF).

Intraosseous meningioma is a rare variant of meningiomas that originates within the cranial bone. Although it can develop in any region of the skull, the most commonly affected areas are the orbital cavity and the frontoparietal region [4]. It has been reported that this variant accounts for only 1% of all intracranial meningiomas [5]. Similarly, other studies have found an incidence of 2.4% among analyzed patients. Due to its low frequency, this variant is associated with limited clinical understanding, diagnostic challenges, and a nonspecific clinical presentation. It is characterized by its intraosseous location and little to no involvement of the dura mater.

However, it is not confined to the cranial convexity; cases have also been reported in areas such as the skull base, paranasal sinuses, and sphenoidal ridge, which may explain visual impairment in some patients [6]. Retrospective reviews have shown that these tumors may occur between 40 and 80 years of age, with an average age of 68.4 years, and a female predominance at a ratio of six women to every three men [7].

Diagnosis and imaging

Diagnostic evaluation of suspected meningioma primarily involves imaging studies and laboratory tests. Butscheidt et al. emphasize laboratory evaluations, particularly measuring serum alkaline phosphatase levels, to help rule out conditions such as Paget's disease [6]. In imaging studies, especially CT, irregularities may be observed in the inner or outer cranial tables, along with signs of hyperostosis due to the tumor’s high affinity for bony structures.

These lesions appear hypodense in approximately 25% of cases or hyperdense in 75% of cases, with calcifications present in 15-20% of patients. Following contrast administration, up to 90% of lesions show enhancement. MRI is also valuable in assessing soft tissue involvement. On MRI, the lesions are typically hyperintense or isointense on T2-weighted sequences and iso- or slightly hypointense on T1. The “dural tail” sign is observed in 52-78% of cases [8].

Surgical management

Surgical resection of the tumor is both diagnostic and therapeutic, aiming for complete resection with wide margins while preserving neurological function. Factors considered include the patient’s age, symptoms, and comorbidities.

Complete resection has been shown to offer a higher chance of cure, lower recurrence rates, and improved survival compared to partial resections. Ideal candidates for tumor resection are patients under 60 years of age, with tumors larger than 25 mm in diameter, without calcifications, no adjacent tissue infiltration, and with lesions in surgically accessible areas [8].

In the study by Harary et al., the importance of reconstructing the bony defect after wide resection was emphasized, with alloplastic materials yielding satisfactory cosmetic outcomes [7]. Traditional mesh systems are adaptable and partly handcrafted, making them cost-effective in emergency contexts. Titanium and polyether ether ketone (PEEK) implants, on the other hand, significantly reduce complication rates in challenging cranial settings. In oncologic cases, inert materials such as titanium or PEEK are preferred, as they allow for better intraoperative handling and provide greater structural stability [9].

In low-resource settings such as ours, single-stage surgical repair is often superior or more practical compared to staged approaches. Performing the procedure in a single session reduces overall costs and shortens the hospital stay, as the patient undergoes only one operative event. Additionally, it minimizes the risk of wound defects that may arise from reinterventions and avoids the need to use surgical equipment and operating room resources multiple times, which is especially important when resources are limited. From the patient’s perspective, single-stage repair is more convenient and comfortable, eliminating the physical and psychological burden of multiple procedures.

## Conclusions

In this case, complete resection was followed by 12 months of disease-free imaging. Larger series consistently report lower recurrence after margin-negative excision, and our experience suggests that, in carefully selected patients, wide resection plus immediate reconstruction can be performed safely. The use of advanced tools, such as craniometry and intra-operative neuronavigation, facilitated precise bony margins in this case, and 3D bone reconstruction enhanced the precision of the procedure.

Additionally, the availability of alloplastic grafts such as PEEK and titanium is advantageous for cranial reconstruction. These materials are preferred over autografts or prosthetic materials such as methyl methacrylate, as they reduce the risk of complications and offer better aesthetic outcomes by being less dependent on the operator’s skill. Whenever possible, our experience suggests that, in carefully selected patients, wide resection plus immediate reconstruction can be performed safely. A single-stage strategy avoided the need for a second anesthetic period in this patient; future series should compare complication profiles between staged and one-stage reconstructions. Titanium mesh provided a satisfactory cosmetic result with no early complications, and the literature suggests similar benefits for other alloplastic materials.

Given that this is a rare case in Nicaragua, with low prevalence and limited awareness among medical professionals, often leading to underdiagnosis, and the first reported case at this institution, this literature review is considered a valuable reference for the diagnostic and therapeutic approach to this pathology in the region.
